# Strengthening health system governance using health facility service charters: a mixed methods assessment of community experiences and perceptions in a district in Kenya

**DOI:** 10.1186/s12913-015-1204-6

**Published:** 2015-12-04

**Authors:** Martin Atela, Pauline Bakibinga, Remare Ettarh, Catherine Kyobutungi, Simon Cohn

**Affiliations:** African Institute for Development Policy, PO Box 14688–00800, Westlands, Nairobi, Kenya; African Population and Health Research Center, PO Box 10787–00100, Nairobi, Kenya; 9236 213th St, NW, Edmonton, AB T5T 4P3 Canada; London School of Hygiene & Tropical Medicine Keppel St, Bloomsbury, London, WC1E 7HT UK

**Keywords:** Facility service charters, Health systems, Accountability, Kenya

## Abstract

**Background:**

Enhancing accountability in health systems is increasingly emphasised as crucial for improving the nature and quality of health service delivery worldwide and particularly in developing countries. Accountability mechanisms include, among others, health facilities committees, suggestion boxes, facility and patient charters. However, there is a dearth of information regarding the nature of and factors that influence the performance of accountability mechanisms, especially in developing countries. We examine community members’ experiences of one such accountability mechanism, the health facility charter in Kericho District, Kenya.

**Methods:**

A household survey was conducted in 2011 among 1,024 respondents (36 % male, 64 % female) aged 17 years and above stratified by health facility catchment area, situated in a division in Kericho District. In addition, sixteen focus group discussions were conducted with health facility users in the four health facility catchment areas. Quantitative data were analysed through frequency distributions and cross-tabulations. Qualitative data were transcribed and analysed using a thematic approach.

**Results:**

The majority (65 %) of household survey respondents had seen their local facility service charter, 84 % of whom had read the information on the charter. Of these, 83 % found the charter to be useful or very useful. According to the respondents, the charters provided useful information about the services offered and their costs, gave users a voice to curb potential overcharging and helped users plan their medical expenses before receiving the service. However, community members cited several challenges with using the charters: non-adherence to charter provisions by health workers; illegibility and language issues; lack of expenditure records; lack of time to read and understand them, often due to pressures around queuing; and socio-cultural limitations.

**Conclusion:**

Findings from this study suggest that improving the compliance of health facilities in districts across Kenya with regard to the implementation of the facility service charter is critical for accountability and community satisfaction with service delivery. To improve the compliance of health facilities, attention needs to be focused on mechanisms that help enforce official guidelines, address capacity gaps, and enhance public awareness of the charters and their use.

## Background

Enhancing accountability in health systems through the use of various mechanisms is increasingly emphasised as crucial for improving the nature and quality of health service delivery worldwide and particularly in developing countries [1]. The overall aim is to increase the responsiveness, sustainability, and efficiency of health services, especially in low and middle-income settings where health systems are struggling to meet the growing challenges of disease burden and shrinking resources [2–7]. Local accountability mechanisms - such as health facilities committees, suggestion boxes, and facility and patient charters – are assumed to provide means for communities to engage directly with local health professionals and hence improve both the perception and provision of health care [3, 8, 9].

Globally, people have become more aware of their rights to health, and hence governments increasingly need to provide meaningful opportunities for individuals to participate in decisions that affect their health and to be answerable on their policy choices and performance [10]. In low and middle income settings in particular, the rising challenges of diseases that were formerly not a threat to populations, such as non-communicable diseases (NCDs) make the need, for engaged citizens who can make informed choices about their lifestyles and how these choices affect their lives, even greater. However, research in accountability and governance in the health sector in many low and middle income settings is generally neglected [10]. It has been argued that facilitating populations to make these choices is not enough; efficient management of competition for policy attention and resources, and attention to reduce the wide disparities in health and in access to health care resources and services is critical [11–13].

In Kenya, like elsewhere, initiatives introduced to enhance accountability and to promote citizens’ participation in decision-making for health at the local level include suggestion boxes, patients’ and facility service charters, customer care desks, health facility committees and hospital boards [8, 9]. However, there is a dearth of information regarding the nature of and factors that influence the performance of such accountability mechanisms, especially in low and middle income countries [14–16]. An exploration of health facility committees in the coastal areas of Kenya highlighted the need to not only improve the training and clarify the roles of health facility committees but also to enhance their interaction with the community [16].

A critical aspect of accountability is the role of health providers in ensuring that people receive the best service possible. A review by Berlan and Shiffman’s [15] identified two major categories of what influences health provider accountability: the health system structure and social influences. Whereas the health system structure refers to the way countries organise, govern and finance health service provision, social influences pertain to factors influencing the way health service providers and consumers think about their roles and indeed view each other [15]. In this paper we focus on health facility service charters to explore the key social influences, namely how providers view accountability and the degree service users have power to influence service provision. Such charters are commitments or undertakings made to various stakeholders about what they can expect in terms of services provided by the facility [17]. They explain what a facility does and how they provide their services and are consequently an important accountability strategy used to promote the rights of patients, ensuring access to equitable and comprehensive health care, promoting the right to choose a care plan, and protect facility users from discrimination [8].

In Kenya, like many other low income settings, data on the use and understanding of service charters are limited [8, 9]. A survey conducted to assess corruption within the public sector showed that the majority (over 90 %) of respondents had never seen a service charter in the public health facility visited [18]. Furthermore, of the 7 % who had seen a service charter, less than 1 % had read the charter, and those who had read it noted that the health service providers did not uphold the charter provisions. Given that Kenya is largely a rural country, and associated health facilities provide the first point of entry for the majority of people into the health system [19–21], this study focuses on the functioning and effectiveness of accountability mechanisms in rural facilities in Kericho District, Kenya. The paper is part of a larger study to map, describe, and analyse factors that influence the performance and effectiveness of health system accountability mechanisms on the delivery of primary health care (PHC) among a rural population in Kericho district, Kenya [22]. Drawing mainly on the qualitative data from the larger project, the focus of this paper is on how useful service charters are in practice as accountability mechanisms for service users.

## Methods

In order to provide a comprehensive account of how health facility charters are received by local users, a mixed methods approach was adopted. Combining a detailed quantitative survey of local users of a sample of facilities with individual qualitative accounts of users’ views and experiences, this study was designed to explore the extent to which local populations were aware of the accountability mechanism, and the extent to which it influenced their experiences when visiting a health centre.

Data came from three sources: i) a household quantitative survey conducted in Kericho district in 2011, through face-to-face interviews with 1,024 respondents; ii) sixteen focus groups with service users; and iii) a facility audit of four purposively selected health facilities, between November 2011 and May 2012. Participants were asked whether or not they had seen a service charter; if they had seen one, whether they had read it; and finally if they had read one, whether or not they felt the information was useful, particularly during their visits to their local health facility.

### Household survey

**Fig. 1:**
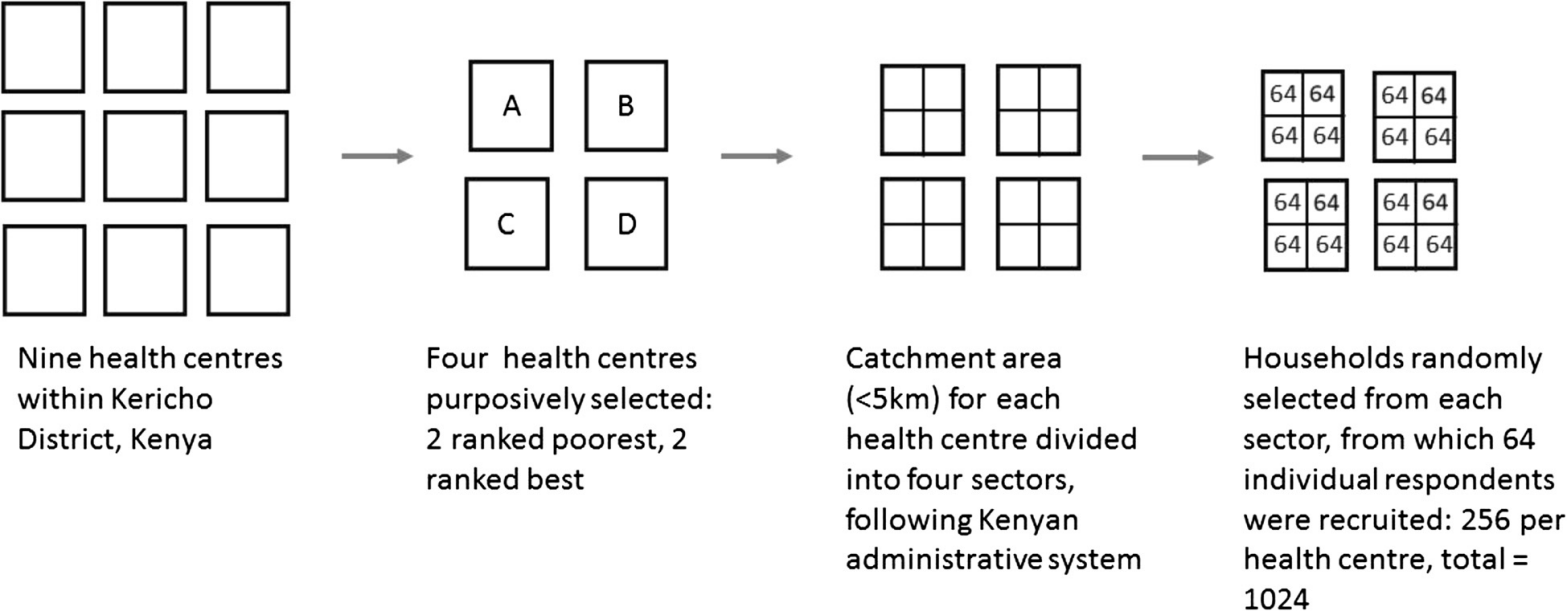
Multi-step sampling strategy for the household survey

The household survey questionnaire was administered using a multi-stage sampling strategy (see Fig. 1). Initially, four facilities were purposefully selected from the 9 health centres in the district based on pilot results and from the district health management team’s ranking on performance; two ranking as poor performers (Facility A [FA] and Facility B [FB]) and two ranking as high performers (Facility C [FC] and Facility D [FD]). A nominal catchment area for each of these (<5 km) was then subdivided into four strata, which were further stratified into 16 stratum according to the existing Kenya administration system. Respondents were selected independently from randomly selected households based on a STATA generated code. The sampling frame was the Kericho District master households’ database obtained from the district statistics office.

These households were then visited on up to three occasions to recruit individual respondents who had resided in the area for at least 6 months and had visited the health centre in the past three months. If, after three visits, a respondent could not be recruited, an alternative household was randomly selected. Recruitment stopped when 64 individual respondents were enrolled for each sector, giving a total of 256 respondents for each health facility, and a total of 1024 respondents.

Table 1 presents a summary of respondent characteristics per facility. The interviews were conducted using a semi-structured questionnaire. The design and wording of the questionnaire was informed by similar quantitative and qualitative work on health system accountability [23–25] and the 2008/09 Kenya Demographic and Health Survey [26].

**Table 1:** Background characteristics of respondents by each health facility

	FA	FB	FC	FD	Total
Individual characteristics					
Number of respondents	256	251	256	256	1019
Sex:					
Male	112 (43.8 %)	88 (35.1 %)	91 (35.5 %)	80 (36.4 %)	371 (36.4 %)
Female	144 (56.3 %)	163 (64.9 %)	165 (64.5 %)	176 (63.6 %)	648 (63.6 %)
Age group:					
17–24 years	85 (33.2 %)	86 (34.3 %)	97 (37.9 %)	811 (31.6 %)	349 (34.2 %)
25–34 years	79 (30.9 %)	62 (24.7 %)	62 (24.2 %)	75 (29.3 %)	278 (27.3 %)
35–44 years	50 (19.5 %)	46 (18.3 %)	46 (18 %)	44 (17.2 %)	186 (18.3 %)
≥ 45 years	42 (16.4 %)	57 (22.7 %)	51 (19.9 %)	56 (21.9 %)	206 (20.2 %)
Current Marital Status:					
Single^a^	96 (37.5 %)	76 (30.3 %)	79 (30.9 %)	72 (28.1 %)	323 (31.7 %)
Married	160 (62.5 %)	175 (69.7 %)	177 (69.1 %)	184 (71.9 %)	696 (68.3 %)
Highest education level:					
Primary school or less	144 (56.3 %)	142 (56.6 %)	143 (55.9 %)	168 (65.6 %)	597 (58.6 %)
Secondary school	92 (35.9 %)	86 (34.3 %)	89 (34.8 %)	77 (30.1 %)	344 (33.8 %)
Post-Secondary	20 (7.8 %)	23 (9.2 %)	24 (9.4 %)	11 (4.3 %)	78 (7.7 %)
Main Occupation					
Agriculture	138 (53.9 %)	163 (64.9 %)	165 (64.5 %)	169 (66 %)	635 (62.3 %)
Skilled Labour	45 (17.6 %)	29 (11.9 %)	31 (12.1 %)	32 (12.5 %)	137 (13.4 %)
Unskilled labour	28 (10.9 %)	21 (8.4 %)	24 (9.4 %)	27 (10.5 %)	100 (9.8 %)
Student	45 (17.6 %)	38 (15.1 %)	36 (14.1 %)	28 (10.9 %)	147 (14.4 %)
Estimated Monthly Income in KES					
≤ 2,000	137 (53.5 %)	117 (46.6 %)	148 (57.8 %)	151 (59 %)	553 (54.3 %)
2001–5,000	79 (30.9 %)	91 (36.3 %)	67 (26.2 %)	65 (25.4 %)	302 (29.6 %)
≥ 5,001	40 (15.6 %)	43 (17.1 %)	41 (16 %)	40 (15.6 %)	164 (16.1 %)
Illness and morbidity					
Ill household member in last facility visit^b^					
≤ 5 Years	64 (25.4 %)	77 (32.9 %)	67 (27.2 %)	83 (33.1 %)	291 (29.6 %)
≥ 5 Years	188 (74.6 %)	157 (67.1 %)	179 (72.8 %)	168 (66.9 %)	693 (70.4 %)
Illness Encountered					
Malaria	119 (47 %)	105 (44.9 %)	98 (39.8 %)	111 (44.2 %)	433 (44 %)
ARI^c^	49 (19.4 %)	80 (34.2 %)	76 (30.9 %)	67 (26.7 %)	272 (27.6 %)
Typhoid	15 (5.9 %)	11 (4.7 %)	9 (3.7 %)	18 (7.2 %)	53 (5.4 %)
Trauma & Accidents	8 (3.2 %)	8 (3.4 %)	15 (6.1 %)	12 (4.8 %)	43 (4.4 %)
NCDs^d^	19 (7.5 %)	7 (3 %)	6 (2.4 %)	1 (0.4 %)	33 (3.4 %)
Others	43 (17 %)	23 (9.8 %)	42 (17.1 %)	42 (16.7 %)	150 (15.2 %)
Treatment Provider					
Health Facility	243 (94.9 %)	220 (87.6 %)	241 (94.1 %)	239 (93.4 %)	943 (92.5 %)
Non-Health Facility	10 (3.9 %)	14 (5.6 %)	5 (2 %)	12 (4.7 %)	41 (4 %)
n/a	3 (1.2 %)	17 (6.8 %)	10 (3.9 %)	5 (2 %)	35 (3.4 %)
Distance to nearest HF					
≤ 3 Km	222 (86.7 %)	180 (71.7 %)	194 (75.8 %)	238 (93 %)	834 (81.8 %)
3–5 Km	21 (8.2 %)	40 (15.9 %)	47 (18.4 %)	1 (0.4 %)	109 (10.7 %)
Don’t Know	13 (5.1 %)	31 (12.4 %)	15 (5.9 %)	17 (6.6 %)	76 (7.5 %)

^a^Includes never married, separated, divorced or widowed

^b^Excludes 35 respondents who did not experience any illness in the 6 months prior to the survey

^c^Acute Respiratory Illness

^d^Non-Communicable Diseases

The questionnaire was administered face-to-face by trained research assistants, to any household member who was at least 17 years old, had lived in the area for at least six months, and lived within 5 km of the health centre. It included questions on respondents’ background (sex, age, education, and marital status), health seeking behaviour, and awareness, perceptions and use of various accountability mechanisms. Most of these assessments were made in the form of structured questions, with some inviting further explanation through supplementary open-ended questions, which constituted a more extensive qualitative interview format. The questionnaire was drafted in English, translated into Kiswahili with the support of a language expert at the African Population and Health Research Center (APHRC) and native speakers, and piloted in exit patient surveys at two facilities in the study district. The two facilities were excluded from the main study. All interviews were conducted in either Kiswahili or English. A few interviews were conducted in the local language – Kipsigis – for those who could not understand either of the two languages.

The study was powered to detect a proportion of at least 40 % of respondents who were aware of accountability mechanisms in the area at 95-percent level of confidence, with a margin of error of 0.04 to enhance the reliability of the results [27] and a design effect of 1.5. The computed sample size per strata was 254 adjusting for non-response. The expected prevalence was based on findings of the pilot study and findings from previous studies in similar context [24, 28] and based on UN recommended sampling strategy for household survey in developing countries [27]. The household survey data were entered using *Sirius*, a software programme to process and manage survey data. The data was checked for logical consistency and coding errors. Analysis was performed using IBM SPSS version 21.

### Focus groups

A total of 16 focus group discussions, consisting of eight to twelve participants, were conducted, (one per stratum). Respondents were selected based on age and gender. Pilot experience revealed that women in this community could not express themselves freely in the presence of their male counterparts for cultural and other reasons. Similarly, young male adults would not freely express themselves in the presence of elders. As a result, and following recommendations from community leaders, focus groups were held separately for males and female grouped into three main age groups: 17–24 years, 25–34 years, and 35 year and above. The discussions were also used to explore further issues that were identified in the survey as being sensitive or polemic [29]. For each facility, specific potential discussion issues were noted from field reports from each research assistant and included in the respective focus groups (Table 2).

**Table 2:** Group composition and discussion guide for the focus group discussions

Age group and gender of focus group participants	Number of participants per gender in each facility				Issues discussed/discussion guide
	FA	FB	FC	FD	
17–24 years					• Barriers to youth/women involvement in health facility management. • Awareness of SC, its functions, and usefulness. • Experiences with using SC members. • Perceived effectiveness/responsiveness of health facilities and health facility committees to youth/women health needs. • Ways to enhance accountability within the HFCs using the service charters
Male		12			
Female		10			
25–34 years					
Male	10				
Female	8				
35 year and above					
Male			10	11	
Female			10	9	

Source: Household Survey April – May 2011

The lead author with the support of note takers guided the focus groups. Discussions were audio-recorded after group consent was obtained, fully transcribed in Kiswahili, translated into English and then checked against the original transcripts. Where translation proved difficult, terms were left in Swahili with accompanying memos in English. These were supplemented by observational notes of each discussion.

The data were analysed using a thematic approach following a path of familiarisation with the data, construction of a preliminary coding scheme, followed by manual qualitative content analysis and interpretation using a method adopted from Graneheim and Lundman [30]. After initial open coding, each code was examined in greater detail for further refinement. Finally, codes were grouped under key themes. Analysis concentrated on the key areas of consensus and disagreement, and, where necessary, on triangulating with other data sources.

### Facility audit

In addition to the qualitative and quantitative approaches above, the study also relied on a facility audit done using a checklist to assess the availability of SCs, the information available on the SCs, including the completeness of the information, location and accessibility of the SCs in accordance with the official guidelines [20, 31–35]. This helped contextualise the information gathered from respondents.

### Ethical approval and informed consent

Approval for the study was obtained from the institutional review committee of APHRC (approval reference HSC/2010/59), the ethics review committees of the Kenya Medical Research Institute (authorization reference KEMRI/RES/7/3/1 PROTOCOL NO.247), and the National Science and Technology Commission (permit number NCST/RR1/12/1/MED/222/4). The research team first visited the households and health facilities sampled for each data collection activity to inform them about the study, deliver a letter of invitation, and make an appointment to conduct the survey when necessary. Village elders and staff of the District Health Management Team were also informed.

Before the start of all interviews, interviewees were read an information sheet explaining the purpose of the research, the institutions involved, the nature of their requested participation, and given the opportunity to ask questions. It was emphasised that the information collected would be confidential and in health facilities, that no individual details would be passed on to district authorities. Written consent was obtained from all interviewees or, where one could not sign, a thumbprint was taken or a nominated close relative signed on their behalf. Consent was also sought specifically for the use of tape recorders during qualitative interviews.

## Results

By adopting various community involvement strategies the study continued until the pre-specified number of respondents for each health facility sector was achieved (*N* = 1,024). However, five questionnaires from respondents in strata FB were discarded due to incomplete information. The analysis presented thus consists of data from 1019 respondents (Table 1).

### Background characteristics of individuals and households

The background characteristics of households and individuals included in the household survey are presented in Table 1. Overall, the survey captured more women (63.6 %) than men (36.4 %), perhaps reflecting the selection criteria requiring one to have used the facility in the last three months. Even though not directly explored in this studies, women have been shown to be more likely to seek and use health care and generally possess greater knowledge about health than men [36, 37]. Moreover, as reported by respondents, it was not uncommon for men in the study community to leave behind their families (wives) to look for jobs in the city. The majority of the respondents (34.2 %) were aged 17–24 years, reflecting the current Kenyan demographic structure [26]. The majority were in a marital relationship (68.3 %). Education levels were generally high for a rural area with 58.6 % having attained primary education. Agriculture (both small scale farming and commercial farming) was the most common occupation practiced by 62.3 % of respondents. Income levels were generally low with more than half (54.3 %) reporting a monthly income of KES 2,000 (approximately $ 24 in 2011) or less, translating to a daily income of less than one US dollar.

### Description of facility service charters (SCs)

According to government policy and guidelines [20, 31–35], a service charter should ideally be placed in a clearly visible place, in most cases at the entrance of the facility. It should be clearly written in visible, legible and client friendly language (for this study area, a Swahili and Kipsigis translation should be available) and it should be updated regularly to reflect any changes in the facility. Additionally, it should contain the names of committee members and where appropriate their phone numbers to enable access by the community whenever they require help from the health facility committees (HFCs). The SC should also contain basic financial information, such as the costs of various services for different categories of patients, the waiting times, facility operation hours, and other relevant health information (ibid). The HFCs should also commit to provide basic facility income and expenditure information as part of their SC.

An audit of the relevant charters revealed that none of these met the basic minimum requirements. Each facility provided varying information and presented it in a different form and location. However, there were some similarities in the SC across all the facilities. These included information about the type of service offered (consultations, lab tests, drugs available), the attendant costs, and the facility working hours. In addition to these, FB had a specific patients’ rights charter within its general service charter, though the print was very small in size and pasted somewhere near the consultation room entrance. Across all the facilities, the information provided was fragmented, incomplete, selective, and in some cases incomprehensible to the users.

### Awareness of service charters

To understand whether this accountability mechanism was relevant/useful to clients, respondents were asked: whether they had seen a charter; if they had, whether they had read it; and if they had, whether they found the information to be useful. Table 3 provides a summary of the results (unweighted). The survey results show that a large proportion (approx. 66 %) of respondents had seen their local facility service charter. The proportion of those who had seen the facility SC was lowest among respondents using FC (50 %) and highest among those using FB (72 %).

**Table 3:** Percent distribution of survey respondents by awareness and perceived service charter ‘Usefulness’ per health facility (un-weighted)

Awareness and use of facility SCs	FA	FB	FC	FD	All facilities
Ever Seen Facility SC	*N* = 251	*N* = 249	*N* = 249	*N* = 247	*N* = 996
Yes	65.7	72.3	50.2	71.3	64.9
No	34.3	27.7	49.8	28.7	35.1
Ever read the information on Facility SC?	*N* = 165	*N* = 180	*N* = 125	*N* = 176	*N* = 646
Yes	82.4	81.1	84.8	87.5	83.9
No	17.6	18.9	15.2	12.5	16.1
How useful do you find the SC	*N* = 143	*N* = 145	*N* = 105	*N* = 152	*N* = 536
Very Useful	9.0	22.8	7.6	5.9	11.6
Useful	82.8	65.5	71.4	65.8	71.1
Less/Not Useful	8.2	11.7	21	28.3	17.4

Respondents provided varying reasons for not knowing about the existence of a service charter for their facility, such as being in a hurry or not bothering to check that such a mechanism existed, while others indicated that it would be difficult to read them while the waiting lines were long and in most cases for care. Others also said that if they stayed to read a charter they risked being seen as idle, or as intending to spread unnecessary *‘fitina’* (petty politics) about the functioning of the facility. There was also a group that argued that one would only notice there was a charter if the facility was performing poorly or if there was a problem. This line of argument was most common among FC users, a facility that was considered one of the best performing in the district, and from which many respondents generally reported satisfaction with its services.

### Perceived usefulness of the service charter

There were varied views about the usefulness of the service charter amongst respondents. For those that had seen one, 84 % read the information. Of this group, 83 % found it useful or very useful in facilitating their interaction with the facility and its management. FB had the highest proportion of respondents who had seen its charter (72 %); but it also had the highest proportion of respondents who had not read the charter (19 %), compared to the average across all facilities of 16 %. FC stands out as having the lowest proportion of respondents who had seen its SC (50 %). Compared to other facilities, FD had the lowest percentage of respondents who reported finding the SC to be very useful. FB had the lowest percentage of respondents reporting finding the SC useful (Table 3). The focus groups provided possible explanations to these variations.

#### Voice to engage health workers and means to curb corruption

Some users who found service charters useful said that it gave them a voice to query charges, if these were more than indicated on the service charter. They argued that even though one may not necessarily confront health workers about overcharging, the fact that the charter contained prescribed costs for services indirectly empowered them to raise the issue. Some focus group discussants also argued that the service charter provided a useful platform to challenge perceived ‘acts of corruption’ and was therefore an important tool for ensuring accountability. The following quotes from some respondents are illustrative:

*It’s not like am saying there is corruption going on here, It’s possible that someone can look at you and charge according to your appearance, but when its openly indicated there, its visible and transparent, you can see it on the board; those who can’t read can ask those who can read to help, but even if they can’t read, it’s something that when someone who can read looks at its good; yes there are those who can’t read, but when they ask say the cost of widal test, they are told its KES 150 ($ 1.81), and if they wish to complain, they can be read for, but it’s much useful to those who can read (Focus group participant FC)*

*The [service charter] can be useful yes, because it would show how they used the money say for last year and this year’s projection will be like this’. It’s good because even those who do not know how to read, they can find someone to read for them. Besides if we had the HFC members’ names and numbers on the board, we would know whom to contact in case of problems (Focus group participant, FA)*

#### Sign of accountability and transparency at the facility

Focus group participants noted that openly displaying important information (about services, the costs, facility working hours, and contact of responsible persons) not only helped them plan their own expenses, but was also a sign of transparency and accountability on the part of the facility management. The majority also reported that the charter symbolised facility management commitment to government standards, since by displaying the information publicly, the government would be aware of what is going on.

#### Source of important information for planning medical expenses

The majority of focus group discussants felt that the charters were useful in providing general but important information, which could for example, enable them plan their medical expenses and help them prepare when they visit the facility. This meant they would know what services are offered, how much to bring with them and to plan their medical expenses ahead, based on the estimates provided:

*Yes, there is a difference, if there is no information displayed, you would not know how much you will pay, but if you see it on the notice board, if they overcharge, you tell them ‘no, let’s go and check the notice board, why are you overcharging and yet here you have indicated a lesser figure? (Male respondent, FC).*

Most also felt that the charter helped people avoid wasting time, for example in facilities where there was no fee exemptions, or where there was a ‘fee-first’ before service policy, respondents felt the charter helped people avoid wasting time by ensuring people carried enough money with them:

*It helps avoid wasting time between the hospital and home because one knows how much they should carry to the hospital (Female Focus group Participant, FA).*

Overall, the service charter was seen to be an important tool to help plan a medical budget, as well as a signifier of transparency at the facility.

### Perceived challenges to using service charters

#### One-sided transparency

Some respondents questioned why the facility charters only provided information about services offered and their associated costs, without revealing expenditure details for the money collected. Focus group participants expressed dissatisfaction with what they saw as one sided-transparency:

*It is confusing because there is no day they [HWs] have told us we have collected this much at the end of the month or the year, we have never heard anything like that, the months and years come and go but we don’t hear about the development in the hospital, yet they keep revising the fees indicated on the notice board. Why can’t they do the same for the monies they’ve collected and the expenditures? Eeeeh…? Why? Something is not right…We can’t know what the money being collected is for, whether it’s for drugs, we just pay and leave (male Focus group participant, FA).*

Although service charters were viewed as being an important accountability mechanism, users felt that this was not sufficient, and that other initiatives, for example educating the community on the need for the fees charged, were necessary. This, they argued, could help reduce tension and enhance cooperation with the health workers, since most community members were actually willing to pay for services or contribute to fund raisers when called upon:

*There is no one to teach/tell us how the money is spent or where it’s taken. For example I have paid KES 100 ($ 1.20), where does it go? The KES 50 ($ 0.60), where does it go? I have paid KES 30 ($ 0.36), where does it go? … I think they should teach the patients when they are outside ‘ this KES 20 ($0.24) goes to what work, this KES 10 ($ 0.12) goes to …’ As it is now, you just pay and go away, so long as you get drugs, so long as you get well (Female Focus group participant FC).*

However, other respondents, especially from facility FC, which was perceived by users to be doing well, were cautious about displaying certain information on notice boards, especially financial information:

*They have receipts and records and there is someone responsible for checking the records. What I know about this place…, one person cannot handle all the work, finances, maintaining the SC, attending to patients, supervising workers…, there are those responsible for collecting money and classifying accordingly e.g. for this and that drug, and we expect that the district hospital [authorities] would follow-up in case of issues, am not sure displaying on that information is useful… (Focus group participant, FC)*

*You know you cannot expose such things, there are things you would not display; there are things you have to put in secret, such as money details (Participant, Male adults focus group, FD).*

#### Health workers’ non-responsiveness, attitude and fear of victimisation

Many respondents reported that even though the charter may provide important information, it was difficult to use such information to engage health workers for fear of victimisation or ‘being marked out’. Discussants indicated that health workers are usually unresponsive to patients’ concerns making them lose hope in relying on the charters for meaningful engagement with the health workers. Many suggested that in such cases, the health workers would simply ignore them, reprimand them, or in some instances deny them service altogether. Previous experiences meant community members had little confidence in raising any issues; in some circumstances even fearing that the drugs they would receive might be compromised because of being ‘too inquisitive’:

*If you start questioning things here they will say you are politicking and I don’t want anything to do with such. In fact there was old man who came here one day, we sat here up to about 11 o’clock and we asked why they were not offering services yet the SC provides that the facility opens at 8 [am] and closes at 5 [pm]. When the old man asked why ‘we are kept waiting from morning without any service’ he was almost chased away; I heard them [HWs] say ‘he wants to interfere with us here’ so I just avoid it because of that politics. I just want to be treated and to go my way (Female Focus group participant, FA).*

#### Social-cultural challenges

Discussants pointed out that the Kipsigis culture did not encourage openness and that in many cases, community issues, including those involving the health facilities, are normally handled by traditional power structures such as the chiefs and the village elders. Moreover, cultural customs dictated that different age groups and genders could only handle facility issues within traditional cultural expectations. This meant that most refrained from directly engaging the health workers in cases where the services received did not match those described in the charter.

*The problem is … as you know our community [the kipsigis] we are not so much exposed, we are not as vocal as other communities, we don’t expose issues, most people will treat these issues [HF issues] as their personal secrets, say a patient if denied drugs [medication] or asked to buy the same outside the facility, they won’t leak out issues, yet the assumption of the SC is an open engagement platform, in fact when you listen to radio, they say it’s your right to ask (village elder FC).*

*The way I know it, our people whenever they have a problem at the facility or elsewhere, they don’t even come to us and at times they go to the district and report to the DMOH, that’s where they raise their matters…eeh eeh eh…it’s a cultural thing, we are not open people (village elder FB).*

#### Non-adherence to facility service charter provisions

The most critical challenge identified by respondents across all the facilities was that in practice the contents of the service charters were frequently not adhered to. Areas identified that undermined the validity or reliability of the charters included the user fees charged, accounting for the facility finances, and waiting and opening times. To corroborate this, a facility audit confirmed that none of the four facilities adhered to its own user fee policy. This was despite the respective HFC setting those charges over and above the official government 10/20 policy, which pegs user fees at public dispensaries and health facilities at KES 20 and KES 10 (approximately 2011 USD 0.24 and 0.12 respectively). The 10/20 policy, announced by the Minister for Health in 2007, was aimed at addressing equity concerns and partly to fulfil a political pledge. Though the policy, the government committed to provide free services for all citizens at dispensaries and health centres, except for a minimum registration fee of KES 10 and KES 20.

In the facilities surveyed, it was not uncommon for clients to be charged more than what was stipulated. All facilities HFCs had set a uniform fee of KES 50 (approximately USD 0.60) for outpatient services. In some cases, patients were forced to pay for services at each of the counters they visited - for instance at the registration desk, consultation desk, facility chemistry, and at the laboratory. They felt helpless and could not question health workers for fear of retribution or being denied service:

*You know the doctor [Health Worker] can say pay this much, as he or she wishes, yeah? Will you argue with the doctor? He says, pay this, you pay… how can you question (with an expression of shock on his face)? If the doctor says, he wants me to pay this amount of money, how can I question what the money is for and I want to get better? All I want is to get better (Male focus group participant, FB).*

## Discussion

This paper has examined the community experiences and perceptions of facility service charters in a rural Kenyan district. A key finding is that none of the charters met the minimum standard set by the government in terms of the information provided. While they all provided a list of services offered and corresponding costs, the information was fragmented. Inconsistencies in the information and what was made available to clients may be attributed to weak government regulation to ensure facilities adhered to official guidelines, or possibly to the fact that most facilities did not have adequate resources to provide a comprehensive structure. As a result it is hard to ascertain what extent what was provided in the charter was ultimately motivated by a desire to be transparent and accountable.

From the study it is clear that awareness of facility charters was relatively high (66 %), suggesting that, with more civic education and enhanced service provider responsiveness, charters can be a useful platform for enhancing accountability and user engagement in health facilities. This finding is contrary to what has been reported elsewhere in Kenya [18] on the low levels of awareness regarding the existence of facility service charters. The comparatively high degree of awareness in this study could be attributed to the high level of literacy in the community, and on-going anti-corruption and health promotion messages broadcast via different media platforms such as radios, televisions and print media.

There were three main ways in which the charter potentially served as a vehicle for accountability. Firstly, it provided users with a voice to curb potential overcharging (though the study found only one case where a user had relied on the SC to query the amount he was charged). Secondly, it provided useful information about the services offered and their costs. Finally, it helped users to plan their medical expenses before coming to the facility for service*.*

However, several challenges experienced by users meant that many did not perceive the service charters as being useful or meet their everyday expectations; these included: lack of adherence to charter provisions by health workers especially in regard to user charges; illegibility and language issues; lack of expenditure records, lack of time to read and understand charter provisions mainly due to long queues, and socio-cultural limitations explain the lack of confidence in the charters as an accountability and engagement mechanism. These findings highlight the limited efficacy of service charters to clients in these settings, mainly due to health system and sociocultural influences, and echo results by other studies in Kenya [18] and other settings outside Kenya [17].

The majority of these issues could be addressed relatively simply: for instance, disseminating comprehensive financial information would satisfy client expectations, since both pilot and survey data revealed the majority of respondents expected this to be the practice, and were disappointed that the facilities did not provide information on how they spent the money they collected.

Displaying such information, however, would require sensitivity, especially in poor areas where the majority still live below poverty line. A number of studies have reported that health workers and administrators were cautious about displaying financial information openly, viewing it as a potential security risk [24, 25]. In addition, maintaining relevant information would require dedicated capacity from staff members, which is not easy given many health workers are already overburdened by the huge number of patients they have to attend to. In fact, one of the major challenges in achieving financial accountability in the facilities described in this paper was staff shortage. For example, most of the facility-in-charges, responsible for overall facility management, also supervise support staff handling finances, yet they have to serve a large number of clients each day. These challenges are also not unique to the study population and have been reported elsewhere [38, 39]. With the new Health Sector Services Fund programme, a new government initiative introduced in 2010 to strengthen health systems through direct funding to PHC facilities currently being implemented [40], there is hope that this level of accountability could be achieved when facilities are provided with professionally trained staff to handle finances.

### Strengths and limitations

Caution needs to be exercised in the interpretation of these findings. First, some of the interviews were conducted in Kipsigis and Kiswahili languages and later translated into English by a language expert and native speakers who are fluent in both languages. As a result, some degree of meaning is inevitably lost in translation. Second, the selection criteria (residence in the area for at least 6 months and within a 5 kilometre radius, use of facility within the last three months before the survey, and being at least 17 years old) limit the findings to this subset of the District’s population. However, communities further than 5 Km were unlikely to use health services in the facilities of interest and hence would not have provided the information needed to assess the effectiveness of the accountability structures without undertaking a huge survey. Third, the sensitivity of the issues may well have encouraged people to give what they thought were socially desirable responses. Nevertheless, by drawing on a large survey dataset derived from a careful sampling strategy, and augmenting this with accounts from face-to-face interviews, focus group discussions, and facility audits, the findings suggest ways in which service charters do not automatically ensure public accountability.

## Conclusion

Improving the compliance of health facilities in districts across Kenya with regard to the implementation of the facility service charter is critical for accountability and community satisfaction with service delivery. Establishing official guidelines on charters without providing the necessary support to ensure that, in practice, they offer the level of transparency intended is unlikely to achieve much. Attention therefore needs to be equally focused on mechanisms to improve government enforcement of official guidelines, addressing capacity gaps in personnel and resources at the facilities, and enhancing public awareness of the charters and their use. In addition, guidelines could include the provision of translations for English versions, training and supervision in the management of costs and expenditure records in health facilities, and the application of a system of sanctions to ensure health workers adhere to charter details.
